# *Arabidopsis* response to low-phosphate conditions includes active changes in actin filaments and PIN2 polarization and is dependent on strigolactone signalling

**DOI:** 10.1093/jxb/eru513

**Published:** 2015-01-21

**Authors:** Manoj Kumar, Nirali Pandya-Kumar, Anandamoy Dam, Hila Haor, Einav Mayzlish-Gati, Eduard Belausov, Smadar Wininger, Mohamad Abu-Abied, Christopher S. P. McErlean, Liam J. Bromhead, Cristina Prandi, Yoram Kapulnik, Hinanit Koltai

**Affiliations:** ^1^Institute of Plant Sciences, Agricultural Research Organization (ARO), the Volcani Center, Bet Dagan 50250, Israel; ^2^School of Chemistry, the University of Sydney, NSW 2006, Australia; ^3^Dipartimento di Chimica, Turin University, 10125 Torino, Italy

**Keywords:** Actin, endocytosis, phosphate, PIN2, root, strigolactone.

## Abstract

Strigolactones (SLs) are plant hormones that regulate the plant response to phosphate (Pi) growth conditions. At least part of SL-signalling execution in roots involves MAX2-dependent effects on PIN2 polar localization in the plasma membrane (PM) and actin bundling and dynamics. We examined PIN2 expression, PIN2 PM localization, endosome trafficking, and actin bundling under low-Pi conditions: a MAX2-dependent reduction in PIN2 trafficking and polarization in the PM, reduced endosome trafficking, and increased actin-filament bundling were detected in root cells. The intracellular protein trafficking that is related to PIN proteins but unassociated with AUX1 PM localization was selectively inhibited. Exogenous supplementation of the synthetic SL GR24 to a SL-deficient mutant (*max4*) led to depletion of PIN2 from the PM under low-Pi conditions. Accordingly, roots of mutants in MAX2, MAX4, PIN2, TIR3, and ACTIN2 showed a reduced low-Pi response compared with the wild type, which could be restored by auxin (for all mutants) or GR24 (for all mutants except *max2-1*). Changes in PIN2 polarity, actin bundling, and vesicle trafficking may be involved in the response to low Pi in roots, dependent on SL/MAX2 signalling.

## Highlight

This study provides an insight into the cellular events of cytoskeleton rearrangement, vesicle trafficking, and PIN protein localization that are associated with low-Pi response and are dependent on strigolactone-auxin cross-talk.

## Introduction

Phosphorus (P) is a vital building block for many essential molecules and macromolecules in the cell and in major metabolic processes in plants. However, soil P availability varies considerably and is considered a growth-limiting factor (Raghothama, 1999). Inorganic phosphate (Pi) is the most readily accessible form of P to plants (Bieleski, 1973), and they have developed several strategies to increase Pi absorption and active uptake. These include structural changes in root architecture and increases in root-hair length and density (Peret *et al.*, 2011).

Regulation of the plant’s response to Pi levels involves the activity of several plant hormones, including auxin. Under conditions of Pi deficiency, auxin signalling leads to changes in root-system architecture, including increased lateral-root formation and decreased primary-root length. Pi-deficient plants are more sensitive to exogenous auxin than Pi-nourished ones (reviewed by Lopez-Bucio *et al.*, 2002; Chiou and Lin, 2011). In addition, the levels of the auxin transporter PIN-FORMED (PIN) proteins in Pi-depleted plants are reduced (González-Mendoza *et al.*, 2013).

The strigolactone (SL) plant hormones affect developmental processes in a number of plant species (Yoneyama *et al.*, 2013). In shoots, SLs suppress the outgrowth of pre-formed axillary buds (e.g. Gomez-Roldan *et al.*, 2008; Umehara *et al.*, 2008), and are positive regulators of secondary-shoot growth (Agusti *et al.*, 2011) and negative regulators of adventitious-root formation (Rasmussen *et al.*, 2012). In roots, SLs regulate lateral-root formation and induce root-hair elongation (Kapulnik *et al.*, 2011). They also regulate primary-root growth by promoting its meristem-cell number (Ruyter-Spira *et al.*, 2011; Koren *et al.*, 2013). In plants, SL is perceived via a specific receptor system that consists of several proteins, including an F-box protein designated MAX2/D3/RMS4, which is linked to a Skp, Cullin, F-box (SCF)-containing complex (reviewed by Waldie *et al.*, 2014; Waters *et al.*, 2014).

SLs may act at least in part by modulating auxin transport. SLs repress shoot branching by enhancing competition between branches, as a result of their ability to reduce the basipetal transport of auxin (Crawford *et al.*, 2010). Accordingly, several studies have demonstrated that SLs influence auxin transport by modulating auxin-efflux PIN activity. In *Arabidopsis*, shoot SLs act by increasing the rate of removal of PIN1 from the plasma membrane (PM) of xylem parenchyma cells in the stem (Shinohara *et al.*, 2013). In the elongation zone of *Arabidopsis* root, during root-hair elongation, at least part of the SL response has been suggested to occur through reduced bundling and induced dynamics of F-actin filaments. Accordingly, PIN2 cellular trafficking and polarity were increased in the PM under the examined conditions (Pandya-Kumar *et al.*, 2014).

In *Arabidopsis* SLs act to regulate, via MAX2-dependent SL signalling, the plant’s perception of or response to Pi conditions (Mayzlish-Gati *et al.*, 2012). The involvement of SLs in the root’s response to low Pi has been also demonstrated in rice (Sun *et al.*, 2014). Here, we showed that, in *Arabidopsis* seedlings, the response to low-Pi conditions involves reduced PIN2 trafficking and polarization in the PM, decreased ARA7-labelled endosome trafficking, and increased actin-filament bundling in root cells. These responses were MAX2-dependent, and exogenous supplementation of the synthetic, biologically active SL GR24 (Johnson *et al.*, 1976; Gomez-Roldan *et al.*, 2008; Umehara *et al.*, 2008) to the SL-deficient mutant (*max4*) led to depletion of PIN2 from the PM under low-Pi conditions. Accordingly, mutants for MAX2, MAX4, PIN2, and TIR3 (required for polar auxin transport) and one ACTIN2 mutant line had a reduced response to low Pi compared with the wild type (WT). This reduced response could be restored by auxin (for all mutants) and GR24 (for all mutants except *max2-1*). Together, these results implicate that increased F-actin bundling and reduced PIN2 levels in the PM are part of an active plant response to low-Pi conditions, and that SLs regulate these cellular responses via MAX2 signalling.

## Materials and methods

### *Arabidopsis* strains, growth conditions, and treatments

Seeds of *Arabidopsis thaliana* used in this study included WT (Col-0 and C24); Col-0 homozygous *max2-1* lines; auxin-transport-deficient and ethylene-insensitive *eir1-1* and *eir1-4*; *TRANSPORT INHIBITOR RESISTANT3* (*TIR3/BIG*) *tir3-103*, *tir3-104*, and *tir3-105* (obtained from the ABRC stock centre; http://abrc.osu.edu/); PIN2::PIN2–GFP (green fluorescent protein) (Vieten *et al.*, 2005; seeds kindly provided by the Department of Molecular Biology & Ecology of Plants, Tel Aviv University, Israel); AUX1::AUX1–YFP (yellow fluorescent protein) (Jones *et al.*, 2009; seeds kindly provided by the Department of Molecular Biology & Ecology of Plants, Tel Aviv University, Israel); 35S::TALIN–GFP (Kost *et al.*, 1998; construct kindly provided by N. H. Chua, The Rockefeller University, NY, USA); 35S::ARA7–GFP (Ueda *et al.*, 2004; construct kindly provided by T. Ueda, University of Tokyo, Japan), and C24 homozygous lines *DEFORMED ROOT HAIR* (DER) *der1-1*, *der1-2*, *der1-3* (obtained from the ABRC stock centre). *max2-1* PIN2::PIN2–GFP-expressing lines (designated *max2-1–22*), *max2-1* 35S::TALIN–GFP-expressing lines (designated *max2-1–28*), and *max2-1*35S::ARA7–GFP expressing lines (designated *max2-1–73*) were identified from F2 progeny of a cross between *max2-1* and Col-0 PIN2::PIN2–GFP, Col-0 35S::TALIN–GFP, and Col-0 35S::ARA7–GFP, respectively, based on F2 and F3 shoot branching and lack of a root-hair elongation response (determined as described by Kapulnik *et al.*, 2011) to GR24. Selected seeds of homozygous lines were used in the present study. *max4-1* PIN2::PIN2–GFP-expressing lines (designated *max4-1–369*) were identified from F2 progeny of a cross between *max4-1* and Col-0 PIN2::PIN2–GFP based on F2 and F3 shoot branching and lack of a root-hair density response.

Seeds were treated as described by Pandya-Kumar *et al.* (2014) and germinated on half-strength MS medium solidified with 0.5% (w/w) Gelzan (Sigma), supplemented with 1.5% (w/v) sucrose and modified to contain 1 µM (for low level) or 2mM (for high level) Pi. Incubation was as described by Pandya-Kumar *et al.* (2014). GR24 (5×10^–5^ M), indole-3-acetic acid (IAA; 5×10^–6^ M), and LatB were applied to the respectively treated seedlings as a 20 µl drop at 0h post-germination (HPG). In all experiments, unless otherwise indicated, for each biological replicate, two Petri dishes were seeded with 10 plants per Petri dish. Three biological replicates were performed for each experiment.

### Determination of root-hair density

The root-hair density of roots grown on GR24 or IAA and control plates was examined as described by Mayzlish-Gati *et al.* (2012), at 48 and 120 HPG Measurements of root-hair density were performed on 10 pictures per treatment, for two 500 µm segments, using IMAGEJ (http://rsbweb.nih.gov/ij/). Experiments were repeated three times; each treatment within each experiment included two replicates. The percentage of change in root-hair density was calculated as: (low Pi – high Pi)/high Pi, (low Pi, GR24 treated – low Pi)/low Pi, or (low Pi, IAA treated – low Pi)/low Pi. Means were subjected to statistical analysis by a Student’s *t*-test or by a one-way analysis of variance (ANOVA) pairwise multiple-comparison Tukey–Kramer test (*P*≤0.05) using the JMP statistical package.

### Live-cell imaging and quantification

The fluorescence intensity of GFP and the endocytic tracer fluorescent styryl dye FM4-64 was examined in live-cell imaging, photographed, and quantified as described by Pandya-Kumar *et al.* (2014). F-actin-filament density was determined according to Nick *et al.* (2009). An eight-pixel-wide probing line was set to integrate the local differences in fluorescence intensity using IMAGEJ. A five-line grid was recorded, and averages for each cell were determined. At least 60 cells were analysed for each treatment and for each measured parameter from at least 30 seedlings grown on high- or low-Pi plates at 48 HPG and 120 HPG. Means of replicates were subjected to Student’s *t*-test (*P*≤0.05) or an ANOVA pairwise multiple-comparison Tukey–Kramer test using the JMP statistical package.

### RNA extraction and quantitative real-time PCR (qPCR)

Gene transcription was examined for Col-0 and *max2-1* genotypes under low- versus high-Pi conditions by qPCR. RNA was extracted as described by Mayzlish-Gati *et al.* (2012) from the lower third of the root of seedlings grown as described above for 48 HPG, using 720 seedlings per sample. cDNA was reverse transcribed from the extracted RNA and subjected to qPCR. The *Arabidopsis* 15S rRNA gene (GenBank accession no. AT1G04270.1) served as the reference gene for the amount of RNA, and was amplified using the specific primers 5′-CAAAGGAGTTGATCTCGATGCTCTT-3′ (forward) and 5′-GCCTCCCTTTTCGCTTTCC-3′ (reverse). To determine PIN2 gene transcription (GenBank accession no. AF086906), the following primers were used: 5′-CTGGTCCAGGTGGAGATGTT-3′ (forward) and 5′-GCCTCCTCTTCCTGCTTTCT-3′ (reverse). qPCR amplification was performed as described by Mayzlish-Gati *et al.* (2010) using the 2^−ΔΔ*C*^_T_ method (Livak and Schmittgen, 2001). The experiment was performed using three biological replicates, with three technical repeats for each. Means±standard error (SE) were calculated for all biological replicates. Values above or below 2 [i.e. that of the numerator (low Pi) versus that of the denominator (high Pi)] represented an increase or decrease, respectively, in the steady-state level of gene transcripts for the examined conditions. Means±SE were calculated for at least three biological replicates for each examined treatment. Means of replicates were subjected to statistical analysis by a Student’s *t-*test (*P*≤0.05) using the JMP statistical package.

## Results

### PIN2 intensity, polarization, and endocytosis are reduced under low-Pi conditions

**Fig. 1.**
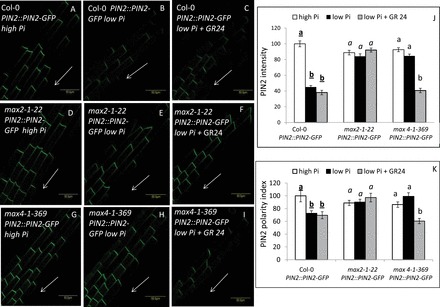
PIN2 PM localization and polarity in the epidermal cells of the primary-root elongation zone in PIN2::PIN2–GFP seedlings grown under high (2mM) and low (1 µM) Pi conditions (48 HPG) and the effect of GR24 treatment. (A–I) PIN2–GFP signal in Col-0 (A–C), *max2-1* (*max2-1–22*; D–F), and *max4-1* (*max4-1–369*; G–I) in roots grown on high-Pi plates (A, D, G), low-Pi plates (B, E, H), and low-Pi plates supplemented with GR24 (C, F, I). Arrows indicate root-wise (downwards). Bars, 50 µm. (J) Intensity of PIN2–GFP signal in the apical plasma membrane of Col-0, *max2-1–22*, and *max4-1–369* roots grown on high-Pi plates (white columns), low-Pi plates (black columns), and low-Pi plates supplemented with GR24 (grey columns). (K) Polarity of the PIN2–GFP signal in the plasma membrane of Col-0, *max2-1–22*, nd *max4-1–369* roots grown on high-Pi plates (white columns), low-Pi plates (black columns), and low-Pi plates supplemented with GR24 (grey columns). Polarity index was determined as the ratio of intensity on the polar versus lateral sides, divided by 2 (Pandya-Kumar *et al.* 2014). Cells (*n*=50–60) from 10 plants were examined for each of three replicates. Different lower-case letters above the bars indicate statistically significant differences between means by a multiple-comparison Tukey–Kramer test (*P*≤0.05).

To examine the effect of low-Pi conditions on PIN2 localization in the PM, WT (Col-0) *Arabidopsis* seedlings expressing PIN2::PIN2–GFP were grown under conditions of low (1 µM) and high (2mM) Pi. PIN2 auxin transporters function in the PM of the epidermal cells of the primary-root elongation zone to maintain cell polarity and auxin balance (Jones *et al.*, 2009; reviewed by Lee and Cho, 2013). PIN2 intensity and polarization (the latter determined as described by Pandya-Kumar *et al.*, 2014, as polarity index, which is the ratio of intensity at the apical versus lateral PM) were found to be reduced under low- compared with high-Pi conditions in the epidermis-cell PM of the root elongation zone at both 48 and 120 HPG (Fig. 1A, B, J, K and Supplementary Fig. S1A, B, E, F, available at *JXB* online). Interestingly, the AUX1–YFP signal in the apical PM of the epidermal cells was significantly elevated under low-Pi compared with high-Pi conditions at 48 HPG (Supplementary Fig. S2, available at *JXB* online).These results suggested that, under the examined conditions, low Pi negatively affects PIN2 but positively affects AUX1 PM localization in epidermal cells in the root elongation zone.

**Fig. 2.**
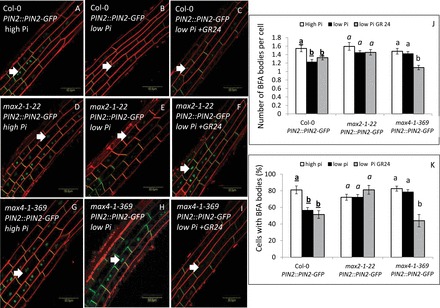
Quantification of PIN2-containing BFA bodies in the epidermal cells of the primary-root elongation zone in PIN2::PIN2–GFP seedlings grown under high (2mM) and low (1 µM) Pi conditions (48h HPG) and the effect of GR24 treatment. (A–I) PIN2-containing BFA bodies (green, PIN–GFP signal; red, FM4-64) in Col-0 (A–C), *max2-1–22* (D–F), and *max4-1–369* (G–I) roots grown on high-Pi plates (A, D, G), low-Pi plates (B, E, H), and low-Pi plates supplemented with GR24 (C, F, I) and treated with BFA (100 µM, 1h). Arrows indicate PIN2-containing BFA bodies. Bars, 50 µm. (J) Number of PIN2-containing BFA bodies per cell in the apical plasma membrane of Col-0, *max2-1–22* and *max4-1–369* roots grown on high-Pi plates (white columns), low-Pi plates (black columns), and low-Pi plates supplemented with GR24 (grey columns) and treated with BFA (100 µM, 1h). (K) Percentage of cells with PIN2-containing BFA bodies in the apical plasma membrane of Col-0, *max2-1–*22, and *max4-1–369* roots grown on high-Pi plates (white columns), low-Pi plates (black columns), and low-Pi plates supplemented with GR24 (grey columns) and treated with BFA (100 µM, 1h). Cells (*n*=50–60) from 10 plants were examined for each of three replicates. Different lower-case letters above the bars indicate statistically significant differences between means by a multiple-comparison Tukey–Kramer test (*P*≤0.05).

PM localization of PIN proteins is determined largely by dynamic vesicle trafficking of constitutive cycling between the PM and the endosomes (e.g. Geldner *et al.*, 2001; Dhonukshe *et al.*, 2007). The vesicle-trafficking inhibitor brefeldin A (BFA) interferes with basal PIN recycling without affecting endocytosis, thereby inducing the accumulation of PIN proteins in ‘BFA bodies’ (Geldner *et al.*, 2001). We examined whether the reduction in PIN2 intensity and polarity in the PM under low-Pi conditions was associated with changes in PIN2 endocytosis. In the WT, the number of PIN2-containing BFA bodies per cell (Fig. 2A, B, J and Supplementary Fig. S3A, B, E, available at *JXB* online) and the percentage of cells with these BFA bodies (Fig. 2A, B, K and Supplementary Fig. S3A, B, F) were reduced under low compared with high-Pi conditions at both 48 and 120 HPG. These results suggested that, under low-Pi conditions, PIN2 intensity, polarization, and endocytosis are reduced.

### Reduction in PIN2 intensity, polarization, and endocytosis under low-Pi conditions at 48 HPG, but not at 120 HPG, is MAX2 dependent

We previously showed that a mutant of SL signalling (*max2-1*) had a reduced root-hair density response to low-Pi conditions relative to the WT at 48 HPG (Mayzlish-Gati *et al.*, 2012). In the present study, we found that, in *max2-1* expressing PIN2::PIN2–GFP, PIN2 intensity, polarity and endocytosis were similar to those recorded under high-Pi conditions in this line at 48 HPG (Figs 1D, E, J, K, and 2D, E, J, K). We also found previously that, at 96 HPG, the root-hair density of *max2-1* on low-Pi plates was similar to that of the WT under the same conditions (Mayzlish-Gati *et al.*, 2012). Accordingly, at 120 HPG, PIN2 intensity and polarity in the PM of *max2-1* were reduced under low- compared with high-Pi conditions, similar to the WT (Supplementary Fig. S1). Moreover, the number of PIN2-containing BFA bodies per cell (Supplementary Fig. S3) and the percentage of cells with these BFA bodies (Supplementary Fig. S3) were reduced in *max2-1* at 120 HPG under low- compared with high-Pi conditions, similar to the WT. These results suggested that the reduction in PIN2 intensity, polarization, and endocytosis under low-Pi conditions at 48 HPG, but not at 120 HPG, is MAX2 dependent.

### The reduction in PIN2 intensity, polarization, and endocytosis under low-Pi conditions at 48 HPG is SL dependent

We previously showed that a mutant of SL biosynthesis (*max4-1*) had a reduced root-hair density response to low-Pi conditions relative to the WT at 48 HPG (Mayzlish-Gati *et al.*, 2012). In the present study, we found that, in *max4-1* expressing PIN2::PIN2–GFP, PIN2 intensity, polarity, and endocytosis were similar to those recorded under high-Pi conditions in this line at 48 HPG (Figs 1G, H, J, K and 2G, H, J, K). These results suggested that the reduction in PIN2 intensity, polarization, and endocytosis under low-Pi conditions at 48 HPG is dependent on SLs.

Moreover, supplementation of GR24 at this time to the seedlings led to depletion of PIN2 from the PM, determined as a reduction in PIN2 intensity and polarization in the PM and reduced endocytosis in *max4-1* but not in *max2-1* or the WT (Figs 1 and 2). Together, these results suggested that SLs are involved in the MAX2-dependent reduction in PIN2 intensity, polarization, and endocytosis under low-Pi conditions at 48 HPG.

### *PIN2* gene expression is not significantly changed under low-Pi conditions in WT and *max2*

To examine whether the recorded reduction in PIN2–GFP signal in the WT under low-Pi conditions involved a reduction in *PIN2* gene expression in the root tips, we determined the level of *PIN2* expression in WT and *max2-1* root tips under low- and high-Pi conditions. As indicated above, *max2-1* PIN2 levels in the PM were not affected by low-Pi conditions. We found that *PIN2* gene expression in the WT under low Pi was 1.54±0.15-fold that under high-Pi conditions. In *max2-1*, *PIN2* expression under low Pi was 1.33±0.3-fold that found under high-Pi conditions. These results suggested only minor changes in *PIN2* gene expression in both WT and *max2-1* under low-Pi compared with high-Pi growth conditions.

### Reduction of endosome movement under low-Pi conditions at 48 HPG is MAX2 dependent

**Fig. 3.**
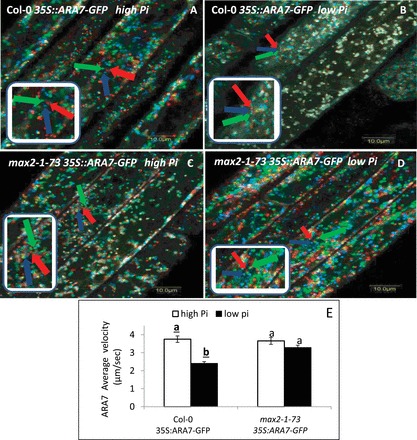
Endosomal movement in the epidermal cells of the primary-root elongation zone in 35S::ARA7–GFP seedlings grown under high (2mM) and low (1 µM) Pi conditions (48 HPG). (A–D) Overlay of frame 1 (red) on frame 5 (+16 s, green), and frame 9 (+29 s, blue) of ARA7-labelled endosomes in seedlings germinated under low-Pi or high-Pi conditions. Bars, 10 µm. (E) Average velocity (µm s^–1^) of ARA7-labelled endosomes in seedlings germinated on low-Pi or high-Pi 48 HPG plates. Different lower-case letters above the bars indicate statistically significant differences between means by a multiple-comparison Tukey–Kramer test (*P*≤0.05). Arrows indicate endosomes at different time intervals [frame 1 (red), frame 5 (+16 s, green), frame 9 (+29 s, blue)].

Since the PM localization of PINs is dependent on endosome trafficking (e.g. Dhonukshe *et al.*, 2007), we examined the effect of low-Pi growth conditions on endosomal movement in epidermal cells of the root elongation zone of WT and *max2-1* by analysing these lines expressing GFP fusions to the endosomal marker ARA7 (Ueda *et al.*, 2004). Endosomal movement was observed as superpositions of a single optical section taken every 3.2 s from the epidermal layer of the roots. The velocity of endosome movement was measured according to Higaki *et al.* (2008). Endosome movement was reduced under low-Pi versus high-Pi conditions at 48 HPG in the WT. This was evident in the overlay of frame 1 (green) on frame 5 (+16 s, red), and on frame 9 (+29 s, blue) of ARA7-labelled endosomes as a reduced proportion of green, red, or blue dots in roots grown under low-Pi conditions (Fig. 3A, B, E). Suggested ‘trails’ of endosomal movement are shown in Fig. 3A and B. Together, these results suggested reduced endosome trafficking under low-Pi conditions in the WT. In *max2-1* expressing 35S::ARA7–GFP, the endosomal velocity under low-Pi conditions was similar to that recorded under high-Pi conditions at 48 HPG (Fig. 3C–E). Together, these results suggested that, under low-Pi conditions at 48 HPG, endosome trafficking is reduced in a MAX2-dependent fashion.

### Bundling of actin filaments increases and their density decreases under low-Pi conditions in a MAX2-dependent fashion

**Fig. 4.**
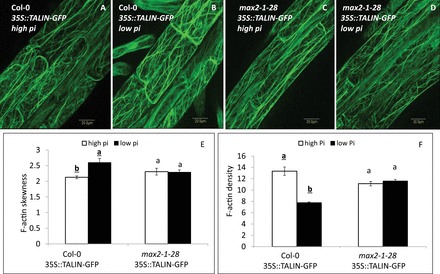
Bundling of TALIN–GFP F-actin in the epidermal cells of the primary-root elongation zone in 35S::TALIN–GFP roots of seedling grown under high (2mM) and low (1 µM) Pi conditions (48 HPG). (A–D) TALIN–GFP signal in Col-0 (A, B) or *max2-1* mutant (*max2-1–28*; C, D) grown under high- or low-Pi conditions. Bars, 20 µm. (E) Skewness of F-actin in Col-0 or *max2-1–28* mutant seedlings grown under high (white columns) or low (black columns) Pi conditions. (F) Density of F-actin in Col-0 or *max2-1–28* mutant seedlings grown under high (white columns) or low (black columns) Pi conditions. Cells (*n*=20–30) from 10 plants were examined for each of three replicates. Different lower-case letters above the bars indicate statistically significant differences between means by a Student’s *t*-test (*P*≤0.05).

PIN2 recycling and vesicle trafficking in the cell are dependent on F-actin in the root epidermal and cortical cells (e.g. Geldner *et al.*, 2001; Kleine-Vehn *et al.*, 2008; Lanza *et al.*, 2012; Nagawa *et al.*, 2012). We therefore examined whether the effect of low Pi on PIN2 localization in the PM of epidermal cells in the primary-root elongation zone was associated with changes in the actin architecture in these cells. Increased bundling and reduced density of actin filaments labelled with TALIN–GFP (Kost *et al.*, 1998) was observed under low- versus high-Pi conditions in WT seedlings at 48 HPG (Fig. 4A, B, E, F). In *max2-1* mutants, no increase in actin-filament bundling or density was recorded at 48 HPG under low- compared with high-Pi conditions (Fig. 4C–F). However, under both low- and high-Pi conditions, the bundling in *max2-1* was slightly (and non-significantly) higher and density was lower than in the WT under high-Pi conditions (Fig. 4).

**Fig. 5.**
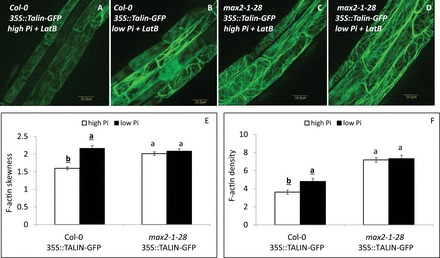
Sensitivity of TALIN–GFP F-actin in the epidermal cells of the primary-root elongation zone to latrunculin B (LatB) treatment in 35S::TALIN–GFP seedlings of Col-0 and *max2-1–28* roots grown under high (2mM) and low (1 µM) Pi conditions (48 HPG) and treated with LatB. (A–D) TALIN–GFP signal in Col-0 (A, B) or *max2-1* mutant (*max2-1–28*; C, D) grown under high- or low-Pi conditions with Lat B treatment. Bars, 20 µm. (E) Skewness of F-actin in Col-0 or *max2-1–28* mutant seedlings grown under high (white columns) or low (black columns) Pi conditions with Lat B treatment. (F) Density of F-actin in Col-0 or *max2-1–28* mutant seedlings grown under high (white columns) or low (black columns) Pi conditions with Lat B treatment. Cells (*n*=20) from seven plants were examined for each of three replicates. Different lower-case letters above the bars indicate statistically significant differences between means by a Student’s *t*-test (*P*≤0.05).

To further show that the effect of low Pi on actin was related to its bundling, we examined F-actin stability in WT and *max2-1* plants. Actin bundling is expected to increase its stability (Staiger and Blanchoin, 2006). To determine actin stability, we tested the effect of latrunculin B (Lat B), which leads to depolymerization of actin filaments (Spector *et al.*, 1983), on actin architecture. Lat B treatment of WT seedlings that developed on high-Pi plates resulted in increased depolymerization of actin in comparison with seedlings developed on low-Pi plates. This was evident as a visible reduction in actin filaments (Fig. 5A, B). It was also evident as a greater reduction—due to Lat B treatment—in F-actin skewness (by 25 and 16%) and density (by 73 and 38%) under high versus low Pi, respectively (Fig. 5E, F) in relation to the LatB non-treated roots (Fig. 4). In *max2-1* at 48 HPG, seedlings grown with both high and low Pi were similarly susceptible to Lat B in terms of its effect on F-actin skewness (by 8 and 9%, respectively) and density (by 35 and 37%, respectively) (Fig. 5C–F) , in relation to the LatB non-treated roots (Fig. 4). Notably, *max2-1* susceptibility to Lat B was reduced relative to that of the WT grown under high-Pi conditions (Fig. 5), in accordance with the results of slightly higher F-actin bundling in *max2-1* than in the WT (Fig. 4). Thus, in a MAX2-dependent fashion, low-Pi conditions lead to increased bundling and reduced density of F-actin relative to high-Pi conditions; this increases actin stabilization and thus actin resistance to depolymerization by Lat B treatment. Together, these results suggested that the increase in actin-filament bundling under low-Pi conditions at 48 HPG is MAX2 dependent.

### Mutants of actin, PIN2, and vesicle trafficking have reduced responses to low-Pi conditions

**Fig. 6.**
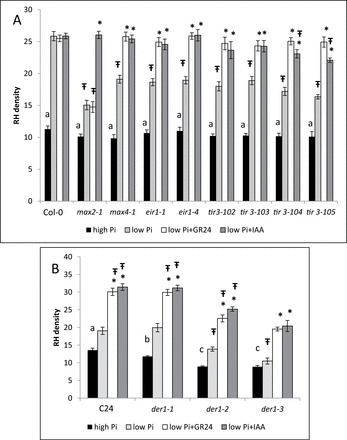
Root-hair density (number per 500 µm) and the effect of GR24 (3×10^–6^ M) and IAA (5×10^–6^ M) treatments on root-hair density in *Arabidopsis thaliana.* (A) WT (Col-0), *max2-1*, *max4-1*, *eir1-1*, *eir 1–4*, *tir3-103*, *tir3-104*, and *tir3-105*. (B) WT (C24), *der1-1*, *der1-2*, and *der1-3.* Seedlings were grown under high (2mM) and low (1 µM) Pi conditions or supplemented under low-Pi conditions with GR24 or IAA (48 HPG) (*n*=300). Means±SE are shown. Asterisks indicate significantly different results between hormone treatments (GR24 or IAA) and non-treated seedlings, all grown under low-Pi conditions, in each of the examined lines. Ŧ indicates that means are significantly different between WT under low-Pi conditions and mutant lines treated with hormones (GR24 or IAA) or non-treated plants, all under low-Pi conditions, as determined by a Student’s *t*-test (*P*≤0.05). Different lower-case letters above the bars indicate statistically significant differences between means of WT and mutant-line seedlings grown under high-Pi conditions by a one-way ANOVA pairwise multiple-comparison Tukey–Kramer test (*P*≤0.05). RH, root hair.

To further examine whether the reduction in PIN2 intensity, polarization, and endocytosis and the increase in actin-filament bundling are necessary for the response to low Pi in *Arabidopsis* seedlings, we examined the response to low Pi of mutants of *ACTIN2* (*ACT2*) (*der1*; Ringli *et al.*, 2002), *PIN2* (*eir1*; e.g. Luschnig *et al.*, 1998; Abas *et al.*, 2006), and the PIN-trafficking-associated protein TRANSPORT INHIBITOR RESISTANT3 (*tir3*; Desgagné-Penix *et al.*, 2005). Their responses were quantified as the level of root-hair density. Mayzlish-Gati *et al.* (2012) showed that root-hair density of *max2-1* (49%) and *max4-1* (94%) is increased under low Pi at 48 HPG but to a lesser extent than that of WT (Col-0, 129%; background of *max2-1*, *max4-1*; Fig. 6A). Similar to the SL mutants, *eir1* lines [*eir1-1* (75%) and *eir1-4* (72%)] and *tir3* mutants [*tir3-102* (76%), *tir3-103* (84%), *tir3-104* (69%), and *tir3-105* (62%)] were less responsive than the WT [Col-0, 129% (background of *eir1* and *tir3* mutant)] to low-Pi conditions at 48 HPG (Fig. 6A). However *der1* lines (*der1-1* [69%] and *der1-2* [55%]) but not *der1-3* (18%) were as responsive as C24 [41%, (background of *der1* mutants)] to low-Pi conditions at 48 HPG (Fig. 6B).

Supplementation of GR24 at 48 HPG, which restores the response of *max4-1* (35%) but not *max2-1* (–2%) to low Pi in terms of root-hair density (Fig. 6A; see also Mayzlish-Gati *et al.*, 2012), effected recovery of the low-Pi response in the *eir1* [*eir1-1* (33%) and *eir1-4* (36%)] and *tir3* mutant lines [*tir3-102* (37%), *tir3-103* (29%), *tir3-104* (46%), and *tir3-105* (52%)] to WT levels (Fig. 6A). Col-0 was not responsive to GR24 under these conditions (Fig. 6A; Mayzlish-Gati *et al.*, 2012), but C24 was (57%; Fig. 6B). The response of most *der* lines [*der1-1* (50%) and *der1-2* (62%)] to GR24 was similar to that of their WT (C24, 57%); the *der1-3* line (86%) also responded significantly to GR24 (Fig. 6B).

Supplementation of IAA at 48 HPG, which restores the response of *max2-1* (73%) to low Pi in terms of root-hair density (Fig. 6A; see also Mayzlish-Gati *et al.*, 2012), enabled recovery of the low-Pi response to WT levels in *max4-1* (33%; see also Mayzlish-Gati *et al.*, 2012) and *eir* lines [*eir1-1* (31%) and *eir1-4* (37%)], and in two of the four *tir* lines [*tir3-102* (31%) and *tir3-103* (28%)] (Fig. 6A). Lines *tir3-104* (34%) and *tir3-105* (35%) also responded significantly to IAA. Col-0 was not responsive to IAA under these conditions (Fig. 6A; see also Mayzlish-Gati *et al.*, 2012); however, C24 was responsive to IAA (64%; Fig. 6B). Here again, the response of two *der* lines [*der1-1* (56%) and *der1-2* (81%)] to IAA was similar to that of their WT (C24, 64%); the *der1-3* line (94%) also responded significantly to IAA (Fig. 6B).

These results suggested that SLs enhance the low-Pi response, determined as increased root-hair density, dependent on MAX2 but independent of PIN2, ACT2, and TIR3, and that auxin enhances the low-Pi response independent of MAX2, MAX4, PIN2, ACT2, and TIR3.

## Discussion

We showed that the plant response to low-Pi conditions is an active process that involves cellular changes in PIN2 trafficking and polarization in the PM, in endosomal movement, and in actin-filament bundling in root cells early during seedling development.

The early response of seedlings to low Pi (e.g. at 48 HPG) is suggested to involve regulation of PIN2 localization in the PM and is SL/MAX2 dependent, based on several lines of evidence. First, reduction in PM localization and polarization of PIN2 was detected in the WT under low- but not high-Pi conditions. Secondly, these reductions were not detected in *max2-1* or *max4-1* mutants at 48 HPG. Accordingly, at that time, *max2-1* and *max4-1* mutants are non-responsive to low Pi (Mayzlish-Gati *et al.*, 2012). Thirdly, in *max4-1*, addition of GR24 led to PIN2 depletion from the PM (this study) and, accordingly, to the low-Pi response (Mayzlish-Gati *et al.*, 2012). Only minor changes in *PIN2* expression were detected under low- compared with high-Pi conditions in both WT and *max2-1*. This suggests that the reduction in PIN2 PM polarity is not a result of changes in *PIN2* gene expression under the examined Pi conditions but rather mainly of changes in PIN2 trafficking under these conditions. Together, these results suggest that SLs are necessary for depletion of PIN2 proteins from the PM of epidermal root cells, and that this depletion is associated with the response to low-Pi conditions in terms of increased root-hair density. Cellular trafficking of AUX1 was shown to be distinct from that of PIN proteins. AUX1 dynamics displayed different sensitivities to trafficking inhibitors compared with PIN1 trafficking and was independent of the endosomal trafficking regulator GNOM ARF-GEF (Kleine-Vehn *et al.*, 2006). Our findings that the AUX1–YFP signal in the PM of the epidermal cells was significantly increased rather than decreased under low-Pi conditions suggest that the intracellular protein trafficking that is related to PIN proteins but unassociated with AUX1 PM localization is selectively inhibited by low-Pi conditions.

Interestingly, at 96 HPG, *max2-1* and *max4-1* mutants are fully responsive to low-Pi conditions (Mayzlish-Gati *et al.*, 2012) and accordingly, in the present study, they had reduced PIN trafficking and PM localization. These results suggest that, under the examined conditions, SLs affect the low-Pi response of reduced PIN trafficking and PM localization mostly at the early stages of development, and that another factor(s), such as other hormonal pathways, dominates later stages.

The polar localization of PINs is determined by the constitutive trafficking of PIN vesicles between the PM and endosomes (e.g. Geldner *et al.*, 2001; Dhonukshe *et al.*, 2007). A significant reduction in the accumulation of PIN2-containing BFA bodies was observed in the WT but not *max2-1* under low-Pi conditions. Since only minor changes in *PIN2* expression were detected under low- versus high-Pi conditions, these results provide additional support for the suggestion that the early response of WT seedlings to low Pi involves regulation of PIN2 localization in the PM and are SL/MAX2 dependent. In agreement with this, González-Mendoza *et al*. (2013) showed that, in the WT, PIN2 levels in the PM decreased under low-Pi conditions, even at 8 and 12 d post-germination. They further showed a reduction in PIN7 levels under low-Pi conditions, suggesting that the reduction in PM distribution is common to different PIN proteins in response to those conditions.

The reduced movement of ARA7-labelled endosomes (Ueda *et al.*, 2004) under low-Pi conditions detected in WT but not in *max2-1* suggests that low-Pi conditions induce a reduction in endosomal trafficking in an SL/MAX2-dependent fashion. Endosomes, including those labelled with ARA7, regulate the recycling, degradation, and localization of different PM proteins (e.g. Reichardt *et al.*, 2007; Reyes *et al.*, 2011). It is therefore likely that the SL/MAX2-dependent response to low Pi involves a general reduction in cellular ARA7-related trafficking, including—but not limited to—that of PIN proteins.

Targeting of the PIN proteins to the PM is largely dependent on F-actin (Geldner *et al.*, 2001; Nagawa *et al.*, 2012). Therefore, the increase in actin bundling observed under low-Pi conditions in the WT might explain the reduction in PIN2 trafficking, PIN2 PM localization, and endosomal movement observed under these conditions in this genotype. Accordingly, neither an increase in actin bundling nor an increase in PIN2 trafficking were detected in *max2-1* mutants at 48 HPG, when they are not responsive to low Pi.

On the one hand, these results further strengthen the association between increased actin bundling and reduced endosome and PIN2 trafficking in response to Pi deficiency. On the other, they suggest that the deficiency in the response of *max2-1* to low-Pi conditions is correlated to its inability to respond to SLs, and that this inability is associated with a lack of induction of changes in actin-filament bundling, endosome and PIN2 trafficking, and PIN2 depletion from the PM. Functional SL/MAX2 signalling may be needed for these cellular events in order to respond to low Pi during the early stages of seedling development.

It should be noted that TALIN–GFP may cause defects in actin organization (Ketelaar *et al.*, 2004) and may therefore complicate interpretation of the results. However, at least in relation to SLs, both TALIN and fABD2-GFP lines (the latter with no detectable adverse effects on actin dynamics or plant morphology; Sheahan *et al.*, 2004) responded similarly (Pandya-Kumar *et al.*, 2014).

Further evidence to support the involvement of PIN2, actin, and vesicle trafficking in the low-Pi response is the reduced low-Pi response in roots of mutants flawed in the activity of PIN2, ACTIN2, and TIR3. Therefore, at early stages of seedling development (e.g. 48 HPG), the response to low Pi involves SL/MAX2-signalling activity, and PIN2, TIR3, and ACTIN2. Restoration of the low-Pi response by GR24 for all mutants except *max2* suggests that SLs can invoke a low-Pi response in roots in an alternative way, which is dependent on MAX2 but not on auxin transport. Indeed, SLs have also been shown to lead to a low-Pi response via induction of TIR1 transcription and thus auxin perception (Mayzlish-Gati *et al.*, 2012). Interestingly, despite the fact that reduction in PIN2 PM localization is necessary for the low-Pi response, *eir1* mutants, flawed in PIN2, exhibit a deficient low-Pi response. It might be that, in the WT, the reduction in PIN2 polarity under low-Pi conditions serves to increase the auxin cellular level in the epidermal root cells, resulting in for example shorter cells and longer root hairs. However, in the *eir1* mutants, as a result of their PIN2 deficiency, auxin flux is disturbed such that auxin maxima needed for execution of a low-Pi response may not be fully formed under the examined low-Pi conditions. The ability of auxin to restore the response of all these mutants to low-Pi conditions may position auxin downstream of the SL/MAX2–actin–PIN2 signal-transduction pathway, further supporting the suggestion that at least one of the mechanisms of SL activity in response to low Pi is based on the regulation of auxin distribution.

Notably, SLs are involved in increased actin bundling and reduced PIN2 polarization under low-Pi conditions (this study). However, their exogenous supplementation reduces actin bundling and increases PIN2 polarization under sufficient-Pi conditions (Pandya-Kumar *et al.*, 2014). In the shoot, as already noted, SLs trigger depletion of the auxin transporter PIN1 from the PM of xylem parenchyma cells (Shinohara *et al.*, 2013), thereby dampening auxin transport (e.g. Crawford *et al.*, 2010; Domagalska and Leyser, 2011). Thus, SLs seem to act to both reduce and enhance PIN distribution in the PM. This dual activity of SLs can be integrated in the model developed by Prusinkiewicz *et al.* (2009) and Shinohara *et al*. (2013), which was built to simulate apical dominance, and suggests the existence of an ‘auxin transport switch’. This ‘switch’ involves feedback between auxin transport by the PIN proteins and auxin flux, and is mediated by the cumulative contribution of a ‘PIN-insertion constant’ and a ‘PIN-removal constant’. It might be that SL/MAX2 act to affect the PIN-removal or PIN-insertion, depending on the growth conditions.

The increase in actin bundling under Pi-deficient conditions and the reduction in PIN2 PM localization and polarization shown here and by González-Mendoza *et al*. (2013) may lead to disturbances in auxin flux. As a consequence, alterations in root development are expected, such as reduced root elongation (González-Mendoza *et al.* 2013) or increased root-hair density (this study), both typical root responses to conditions of Pi deficiency (Peret *et al.*, 2011).

Interestingly, various other stress acclimations and responses in plants involve regulation of cytoskeleton structure and dynamics, and vesicle trafficking. For example, plants acquire freezing tolerance through cold acclimatization, while actin re-organization serves as a link between membrane rigidification and calcium influx necessary for this acclimation (Örvar *et al.*, 2000). An increase in actin bundling has also been detected following short-term salt treatment, and has been suggested to play a vital role in salt and osmotic stress tolerance in *Arabidopsis* (Wang *et al*., 2010). Aluminium interferes with FM4-64 internalization and inhibits the formation of BFA-induced compartments in root cells (Illéš *et al.*, 2006). Regulators of membrane traffic, RABA1 members, which mediate transport between the *trans*-Golgi network and the PM, were found to be required for salinity stress tolerance (Asaoka *et al.*, 2013). Also, acceleration of endocytosis by overexpression of AtRabG3e, a vesicle trafficking-regulating gene, led to increased tolerance to ionic (salt) or osmotic (sorbitol) stress conditions (Mazel *et al.*, 2004).

In addition, several stress responses are associated with changes in auxin transport. For example, changes in auxin transport affect plant tolerance to arsenite, salinity, and high-temperature stress conditions (Krishnamurthy and Rathinasabapathi, 2013). More specifically, reactive oxygen species produced during different stress conditions can have an impact on the auxin redistribution via repression of the polar auxin transporters (reviewed by Tognetti *et al.*, 2012). However, the reactive oxygen species effect on auxin efflux may also result from the induction, by stress conditions, of flavonoid accumulation, which negatively affects auxin transport by other mechanisms (Murphy *et al.*, 2000; Peer *et al.*, 2013). Also, cold stress was shown primarily to target intracellular auxin transport, by selectively inhibiting the intracellular trafficking (monitored by ARA7–GFP movement) and polar localization of PIN3 (Shibasaki *et al.*, 2009). It was suggested that the cold stress reduced intracellular cycling affects the functionality of PIN, which may result in a reduction of shootward transport of auxin (Shibasaki *et al.*, 2009). Interestingly, similar to our results, cold stress selectively inhibited the intracellular protein trafficking that was related to PIN proteins and not that related to other membrane proteins. However, in this case of cold stress, actin played only a limited role in suppressing the trafficking of PIN2 and ARA7 in *Arabidopsis* root (Shibasaki *et al.* 2009).

Taken together, changes in actin architecture and/or vesicle trafficking and PIN PM localization underlie various stress responses in plants. Since the recorded changes in PIN2 polarity, actin bundling, and vesicle trafficking in the response to low Pi are suggested to be dependent on SL/MAX2 signalling, it might be interesting to examine the involvement of the SL/MAX2- dependent cellular events in plant responses to other abiotic stress conditions.

## Supplementary data

Supplementary data are available at *JXB* online.

Fig. S1. PIN2 plasma membrane localization and polarity in epidermal cells of the primary-root elongation zone in PIN2::PIN2–GFP seedlings grown under high (2mM) and low (1 µM) phosphate (Pi) conditions (120h post-germination).

Fig. S2. AUX1 plasma membrane localization and polarity in epidermal cells of the primary-root elongation zone in AUX1::AUX1-YFP seedlings grown under high (2mM) and low (1 µM) phosphate (Pi) conditions (48h post-germination).

Fig. S3. Quantification of PIN2-containing BFA bodies in the epidermal cells of the primary-root elongation zone in PIN2::PIN2–GFP seedlings grown under high (2mM) and low (1 µM) phosphate (Pi) conditions (120h post-germination).

Supplementary Data

## Abbreviations:

ANOVA: analysis of variance
GFP: green fluorescent protein
IAA: indole-3-acetic acid
PM: plasma membrane
P: phosphorus
Pi: inorganic phosphate
qPCR: quantitative real-time PCR
SE: standard error
SL: strigolactone
WT: wild type
YFP: yellow fluorescent protein.
