# Supplementary material for: Arabidopsis response to low-phosphate conditions includes active changes in actin filaments and PIN2 polarization and is dependent on strigolactone signalling
Source: J Exp Bot. 2015 Jan 21;66(5):1499–510. doi: 10.1093/jxb/eru513 (PMC4339606; doi:10.1093/jxb/eru513)
Supplement: Supplementary Data [file supp_66_5_1499__index.html]

 Arabidopsis response to low-phosphate conditions includes active changes in actin filaments and PIN2 polarization and is dependent on strigolactone signalling — Arabidopsis response to low-phosphate conditions includes active changes in actin filaments and PIN2 polarization and is dependent on strigolactone signalling — Supplementary Data 

# *Arabidopsis* response to low-phosphate conditions includes active changes in actin filaments and PIN2 polarization and is dependent on strigolactone signalling

## Supplementary Data

Data files

**Files in this Data Supplement:**

- Supplementary Data - Supplementary Data
