# Supplementary material for: Arabidopsis response to low-phosphate conditions includes active changes in actin filaments and PIN2 polarization and is dependent on strigolactone signalling
Source: J Exp Bot. 2015 Jan 21;66(5):1499–510. doi: 10.1093/jxb/eru513 (PMC4339606; doi:10.1093/jxb/eru513)
Supplement: Supplementary Data [file supp_eru513_jexbot137166_file001.pdf]

Supplementary Data

Arabidopsis Response to Low-Phosphate Conditions Includes Active Changes in Actin Filaments and PIN2 Polarization and Is Dependent on Strigolactone Signaling

Manoj Kumar,[1†] Nirali Pandya-Kumar,[1†] Anandamoy Dam,[1] Hila Haor, [1] Einav Mayzlish-Gati,[1] Eduard Belausov,[1] Smadar Wininger,[1] Mohamad Abu-Abied,[1] Christopher S. P. McErlean,[2] Liam J. Bromhead,[2] Cristina Prandi,[3] Yoram Kapulnik,[1] and Hinanit Koltai[1,]*

Figure legends

Figure S1. PIN2 plasma-membrane localization and polarity in epidermal cells of the primary-root elongation zone in PIN2::PIN2–GFP seedlings grown under high (2 mM) and low (1 µM) phosphate (Pi) conditions (120 h postgermination).

(A–D) PIN2–GFP signal in roots of Col-0 (A, B) and *max2-1* (*max2-1-22*; C, D) seedlings grown on high-Pi plates (A, C) and low-Pi plates (B, D) (scale bars = 50 µm).

(E) Intensity of the PIN2–GFP signal in the apical plasma membrane of Col-0 and *max2-1-22* roots grown on high-Pi plates (white columns) and low-Pi plates (black columns).

(F) Polarity of the PIN2-GFP signal in the plasma membrane of Col-0 and *max2-1-22* roots grown on high-Pi plates (white columns) and low-Pi plates (black columns). Polarity index was determined as the ratio of intensity on the polar versus lateral sides, divided by 2 (Dhonukshe *et al.*, 2008).

Cells (n = 50–60) from 10 plants were examined per each of three replicates. Different lowercase letters above the bars indicate statistically significant differences between means by one-way ANOVA pairwise Student's t-test ($P \leq 0.05$). Arrows point toward the roots.

Figure S2. AUX1 plasma-membrane localization in epidermal cells of the primary-root elongation zone in AUX1::AUX1–YFP seedlings grown under high (2 mM) and low (1 µM) phosphate (Pi) conditions (48 h postgermination).

(A–B) AUX1–YFP signal in roots of Col-0 (A, B) seedlings grown on high-Pi plates (A) and low-Pi plates (B) (scale bars = 50 µm).

(C) Intensity of the AUX1–YFP signal in the apical plasma membrane of Col-0 roots grown on high-Pi plates (white columns) and low-Pi plates (black columns).

Cells (n = 50–60) from 10 plants were examined per each of three replicates. Different lowercase letters above the bars indicate statistically significant differences between means by one-way ANOVA pairwise Student's t-test ($P \leq 0.05$). Arrows point toward the roots.

Figure S3. Quantification of PIN2-containing BFA bodies in the epidermal cells of the primary-root elongation zone in PIN2::PIN2–GFP seedlings grown under high (2 mM) and low (1 µM) phosphate (Pi) conditions (120 h postgermination)

(A–D) PIN2-containing BFA bodies (green – PIN–GFP signal; red – FM4-64) in Col-0 (A, B) and *max2-1-22* (C, D) roots grown on high-Pi plates (A, C) and low-Pi plates (B, D) and treated with BFA (100 µM, 1 h) (scale bars = 50 µm).

(E) Number of PIN2-containing BFA bodies per cell in the apical plasma membrane of Col-0 and *max2-1-22* roots grown on high-Pi plates (white columns) and low-Pi plates (black columns) and treated with BFA (100 µM, 1 h).

(F) Percentage of cells with PIN2-containing BFA bodies in the apical plasma membrane of Col-0 and *max2-1-22* roots grown on high-Pi plates (white columns) and low-Pi plates (black columns) and treated with BFA (100 µM, 1 h).

Cells (n = 50–60) from 10 plants were examined per each of three replicates. Different lowercase letters above the bars indicate statistically significant differences between means by one-way ANOVA pairwise Student's t-test ($P \leq 0.05$). Arrows indicate PIN2-containing BFA bodies.

Figure S1.

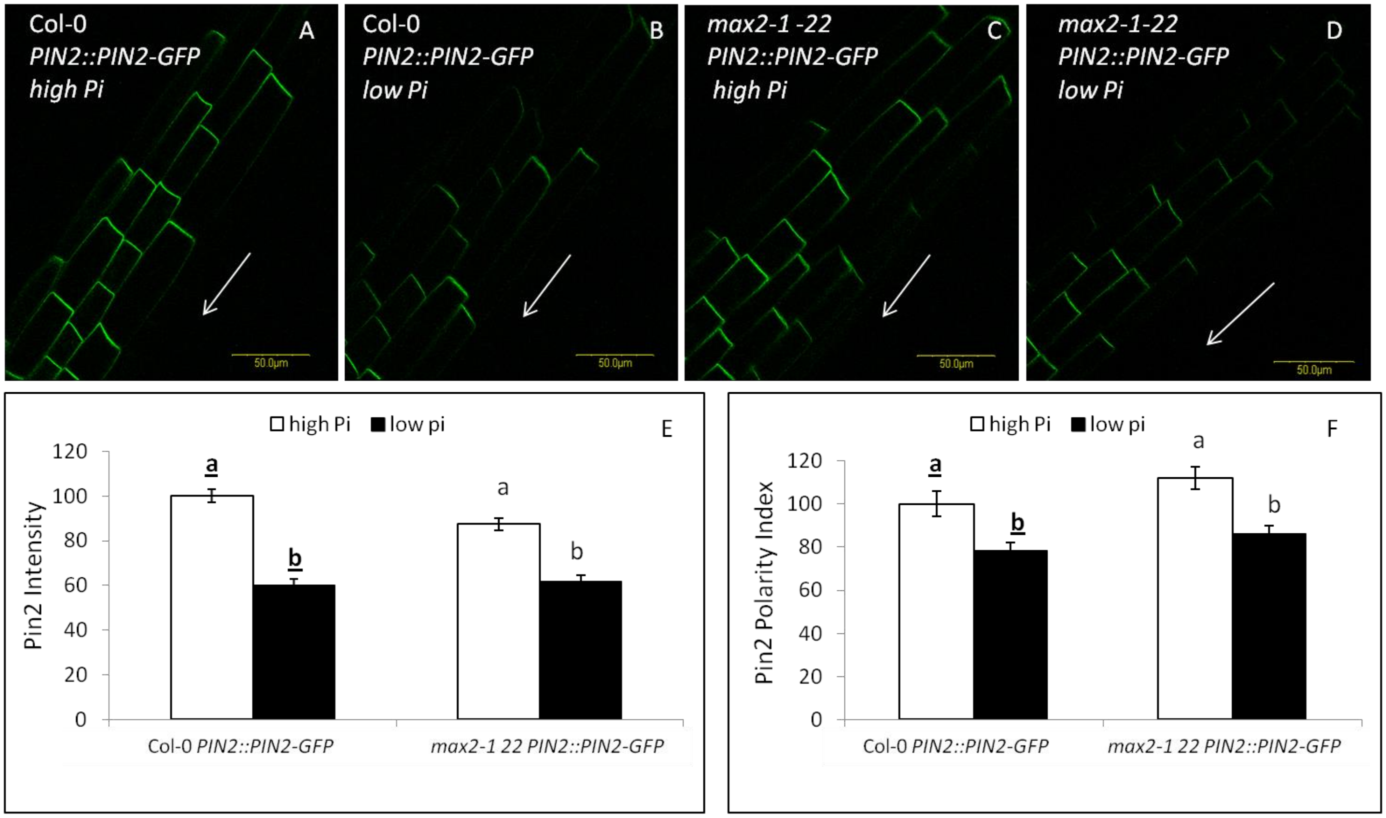
Col-0
PIN2::PIN2-GFP
high Pi
A
50.0µm
Col-0
PIN2::PIN2-GFP
low Pi
B
50.0µm
max2-1 -22
PIN2::PIN2-GFP
high Pi
C
50.0µm
max2-1-22
PIN2::PIN2-GFP
low Pi
D
50.0µm
high Pi
low pi
E
Pin2 Intensity
120
100
80
60
40
20
0
a
b
a
b
Col-0 PIN2::PIN2-GFP
max2-1 22 PIN2::PIN2-GFP
high Pi
low pi
F
Pin2 Polarity Index
120
100
80
60
40
20
0
a
b
a
b
Col-0 PIN2::PIN2-GFP
max2-1 22 PIN2::PIN2-GFP

Figure S2.

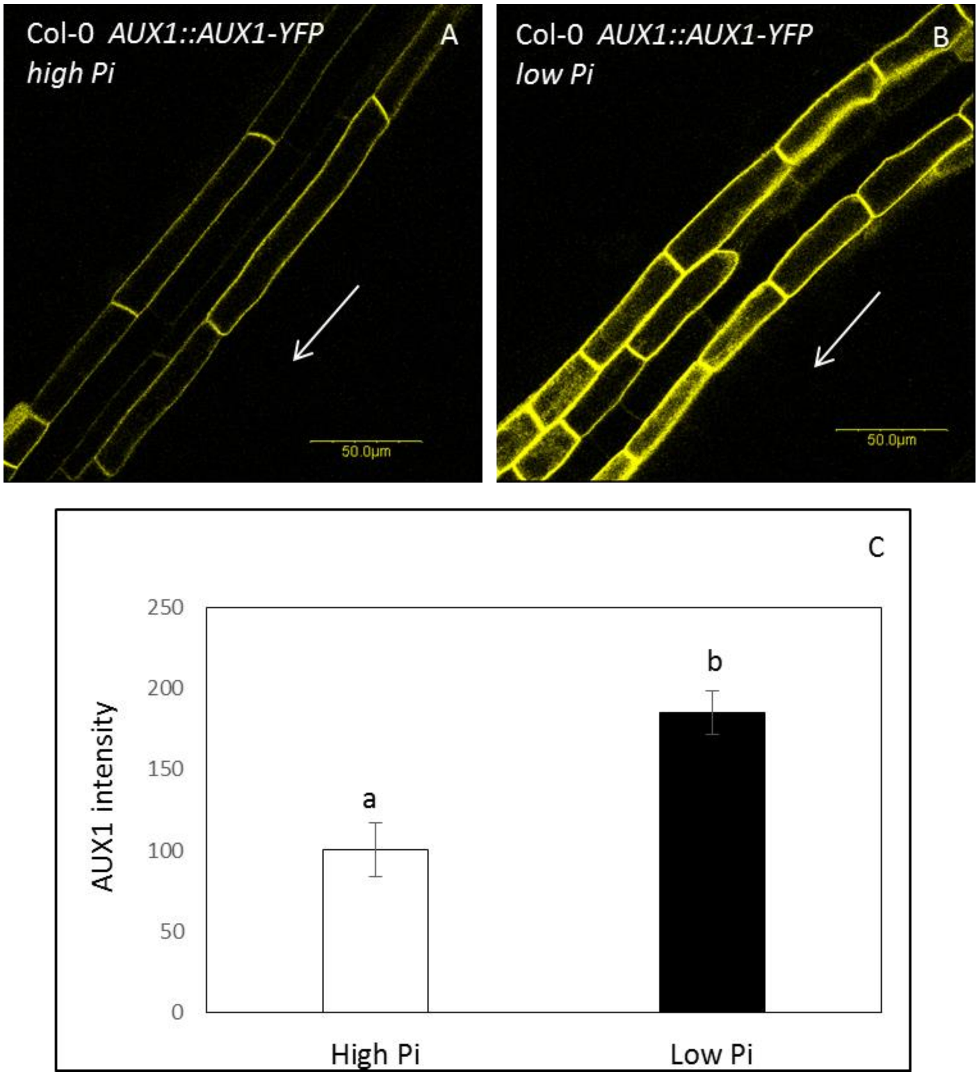
Col-0 AUX1::AUX1-YFP
high Pi
A
50.0µm
Col-0 AUX1::AUX1-YFP
low Pi
B
50.0µm
C
250
200
150
100
50
0
AUX1 intensity
a
b
High Pi
Low Pi

Figure S3.

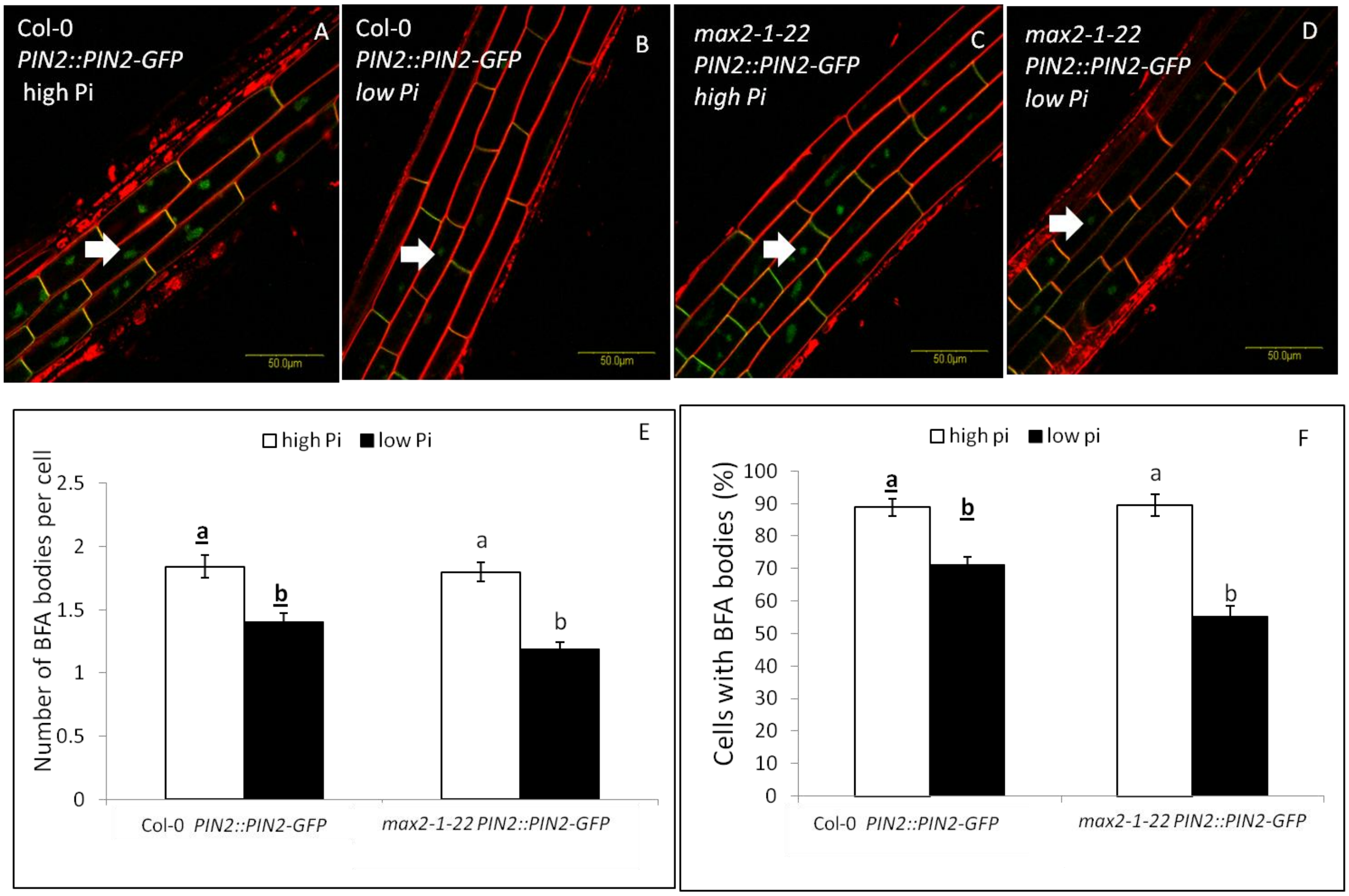

Col-0
PIN2::PIN2-GFP
high Pi
A
50.0µm
Col-0
PIN2::PIN2-GFP
low Pi
B
50.0µm
max2-1-22
PIN2::PIN2-GFP
high Pi
C
50.0µm
max2-1-22
PIN2::PIN2-GFP
low Pi
D
50.0µm
high Pi
low Pi
E
Number of BFA bodies per cell
2.5
2
1.5
1
0.5
0
a
b
a
b
Col-0 PIN2::PIN2-GFP
max2-1-22 PIN2::PIN2-GFP
high pi
low pi
F
Cells with BFA bodies (%)
100
90
80
70
60
50
40
30
20
10
0
a
b
a
b
Col-0 PIN2::PIN2-GFP
max2-1-22 PIN2::PIN2-GFP
